# The Hospitals of Our Grandfathers

**Published:** 1886-10-30

**Authors:** 


### THE HOSPITALS OF OUR GRANDFATHERS.

(*Continued from page* 40.)

An Old Paris Hospital.

In 1693, it is said that at the *Hôtel Dieu* twelve or fifteen patients shared the same bed—that is to say, some lay on the bed and some under it, in turns. In 1709 as many as 9,000 patients are said to have been sheltered in this Parisian hospital. Where the passages into which the wards opened were narrow, the air became stagnant and suffocating, and patients, partially clad, might often have been seen on the bridge of St. Charles, which connected two portions of the hospital across the Seine, whither they had hurried for a little fresh air, from out of the stifling heat and stench of their beds. In many parts of the place the wards were badly lighted, and it is easy to understand that patients might often die some time before the fact was discovered. The living and the dead are said frequently to have been seen there lying side by side in the same bed. The convalescent ward was on the third story, and could be reached only by passing through a room devoted to small-pox patients. The insane ward was next to one filled with sufferers who had recently undergone the most painful operations, and who were constantly disturbed by the cries of their mad neighbours, at the very time when, of course, they were feeble and exhausted, and their nerves unstrung.

A Dreadful Picture.

The operating theatre was often filled with those who had undergone, were undergoing, or were about to undergo, operations,—and that, be it remembered, at a time when anæsthetics were wholly unknown. Just imagine for a moment the incessant shrieking and the frantic prayers, the cries and curses that from some corner or other of that dreadful building, with its thousands of occupants, must have been wailing out all day long, and think of the shuddering terror of the poor wretches whose turn was about to come. One ward was reserved for pregnant women—the virtuous and the vile, the healthy and the diseased, indiscriminately mingled together; and it frequently happened that contagious and non-contagious maladies were brought together in the same ward—one patient with small-pox and another with fever lying side by side. Apart from all considerations of humanity, it was, of course, a horribly unwholesome state of things, and it is not surprising that a pestilence swept through the place like a hurricane.

Old London Hospitals.

We do not appear ever to have had anything quite so bad as that in London, not, at any rate, on such a scale. At the time referred to, our large institutions for the sick and suffering, for the most part, were by comparison models of all that such places should be, so far as the medical science of the day could make them. But it is amazing to find even in them evidence of a want of knowledge of many things that in our day would strike even the unprofessional mind as the merest elements of hygiene. Everybody of the smallest intelligence knows how important a matter the ventilation of hospital wards is held to be. In the best appointed institutions not only is the utmost care taken to constantly renew the atmosphere of a ward, but the system of renewal is so arranged that, so far as it is possible to avoid it, there shall be no currents of air moving from patient to patient across the beds. A century ago arrangements for ventilation of any effective kind appear to have been quite the exception, even in the best of our London hospitals. Apertures over the doors contributed the only provision of the sort in one of the best managed of them, and an experienced observer, in going through many of the rest, refers to this as an excellent feature for imitation as a means of mitigating the closeness and foulness of the air in the wards in which sick people were supposed to be battling with disease, and regaining health and strength. It need hardly be said that, at that time, some of the more obscure of the continental hospitals were in a sad plight. At Lyons, the air which patients were breathing was foul and poisonous in the extreme, and at Malta it is said that the beds were in such a filthy condition that they had to be perfumed, and that even then the doctors never came near them without holding their handkerchiefs to their faces. We are so apt to take things for granted nowadays, that it cannot be unprofitable to recall some of the evils from which our own generation is happily free. The voice of criticism would often be hushed if the speaker could compare what was with what now is.

